# Cerebral Large Vessel Occlusion Caused by Fat Embolism—A Case Series and Review of the Literature

**DOI:** 10.3389/fneur.2021.746099

**Published:** 2021-10-13

**Authors:** Suyi Ooi, Subahari Raviskanthan, Bruce C. V. Campbell, Elspeth J. Hutton, Peter J. Mitchell, Geoffrey C. Cloud

**Affiliations:** ^1^Department of Neurology, Royal Melbourne Hospital, Melbourne, VIC, Australia; ^2^Department of Neurology, Monash Health, Melbourne, VIC, Australia; ^3^Department of Neurology, Alfred Health, Melbourne, VIC, Australia; ^4^Department of Medicine, University of Melbourne, Melbourne, VIC, Australia; ^5^Department of Neuroscience, Monash University, Melbourne, VIC, Australia; ^6^Department of Radiology, Royal Melbourne Hospital, University of Melbourne, Melbourne, VIC, Australia

**Keywords:** ischaemic stroke, large vessel occlusion (LVO), fat embolism, surgical complication, endovascular clot retrieval

## Abstract

The diagnosis of fat embolism syndrome typically involves neurological, respiratory and dermatological manifestations of microvascular occlusion 24–72 h after a precipitating event. However, fat embolism causing cerebral large vessel occlusion strokes and their sequelae have rarely been reported in the literature. This case series reports three patients with fat emboli post operatively causing cerebral large vessel occlusions, as well as a review of the literature to identify differences in clinical presentations and outcomes in stroke secondary to fat emboli causing large vessel occlusions compared to those with fat embolism syndrome.

## Case 1

**Figure 1:**
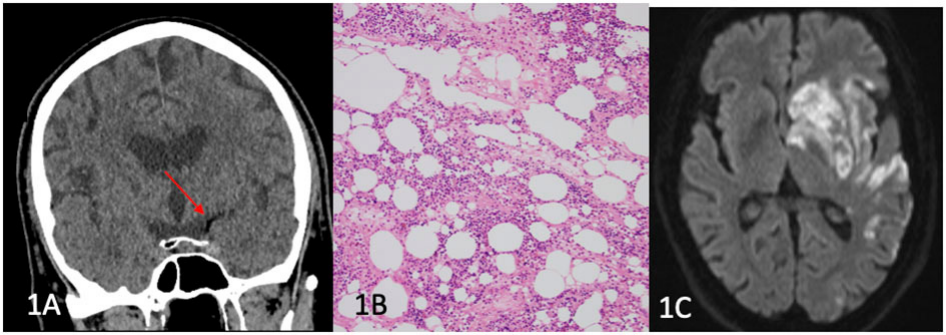
**(A)** Left hypodense M1 artery (red arrow), **(B)** histopathology showing bone marrow constituents and adipose tissue consistent with fat embolism, **(C)** Diffusion weighted imaging (DWI) showing left MCA territory infarction.

A 71-year-old man with a history of hypertension, hypercholesterolaemia and osteoarthritis, underwent an elective left knee replacement lasting 150 min under general and spinal anaesthetic, using a limb tourniquet. Post-operatively, he was found unresponsive with a Glasgow Coma Score (GCS) of 6 (E1V2M3) and had a global aphasia and right hemiparesis with an initial National Institutes of Health Stroke Score (NIHSS) of 24. CT brain (Figure 1A) demonstrated a “hypodense artery sign” with Hounsfield units (HU) of −75 in the left M1 segment of the middle cerebral artery (MCA) suggestive of fat embolism, and tandem occlusions in the left internal carotid artery (ICA) and M1 MCA were seen on CT angiogram (CT-A).

He underwent endovascular clot retrieval (ECR) and reperfusion was achieved with large volume clot aspirated from the left M1 and M2 segments. Histopathology of the aspirated clot revealed adipose tissue and bone marrow with red blood cells, white cells and platelets, and fibrin fragments containing red blood cells with an admixture of eosinophils and neutrophils (Figure 1B). Magnetic resonance imaging (MRI) of the brain performed at day 3 (Figure 1C) showed extensive left MCA territory infarct with haemorrhagic transformation within the left basal ganglia. Transoesophageal echocardiogram revealed a large patent foramen ovale (PFO). At day 60, he had ongoing global aphasia, left sided gaze preference and dense right hemiparesis.

## Case 2

**Figure 2:**
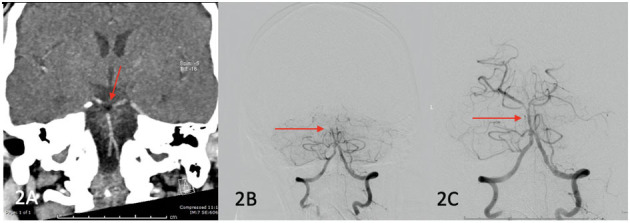
**(A)** (Red arrow) fat embolism demonstrated in the basilar artery, **(B)** pre recanalization ECR images confirming occlusive thrombus in basilar artery (red arrow), **(C)** post recanalization images showing reperfusion of mid and distal basilar artery (red arrow).

A 47-year-old male with a background of valvular atrial fibrillation on warfarin, type 2 diabetes mellitus, chronic obstructive pulmonary disease, stage 3 chronic kidney disease and obesity was admitted for a mechanical mitral valve replacement for rheumatic mitral valve disease. Intraoperative cross-clamping time and bypass time was 66 and 90 min, respectively. Forty-eight hours post operatively, he developed evolving right sided hemiparesis and facial weakness, right gaze preference and right homonymous inferior hemianopia. CT-B initially did not demonstrate any acute changes although a hypodense basilar was noted retrospectively. At day 5 post-sternotomy, he had a deterioration in conscious state from GCS 15 to GCS 10 (E3V1M6). CT-A demonstrated a hypodense basilar artery (HU of−44) extending into the posterior cerebral arteries bilaterally (Figure 2A). He proceeded to ECR with successful recanalisation and stenting of the mid-basilar artery (see Figures 2A–C). Repeat CT-B showed established bilateral occipital and cerebellar strokes. The patient was managed with 48 h of heparin infusion post thrombectomy after which warfarin was reinstated. PFO was not demonstrated on post-operative TTE. He made a steady recovery and at 12 months post stroke was able to ambulate 50 metres without a gait aid.

## Case 3

A 69-year-old female with a past medical history of mastocytosis, asthma, and previous provoked deep venous thrombosis presented after a mechanical fall, sustaining a comminuted left intertrochanteric femoral fracture. She underwent insertion of a left intramedullary nail the next day, positioned in the right lateral position. Intraoperatively, concerns about fat embolism were raised after a transient period of hypoxia and hypotension that occurred 5 min after completion of the intramedullary nailing.

**Figure 3:**
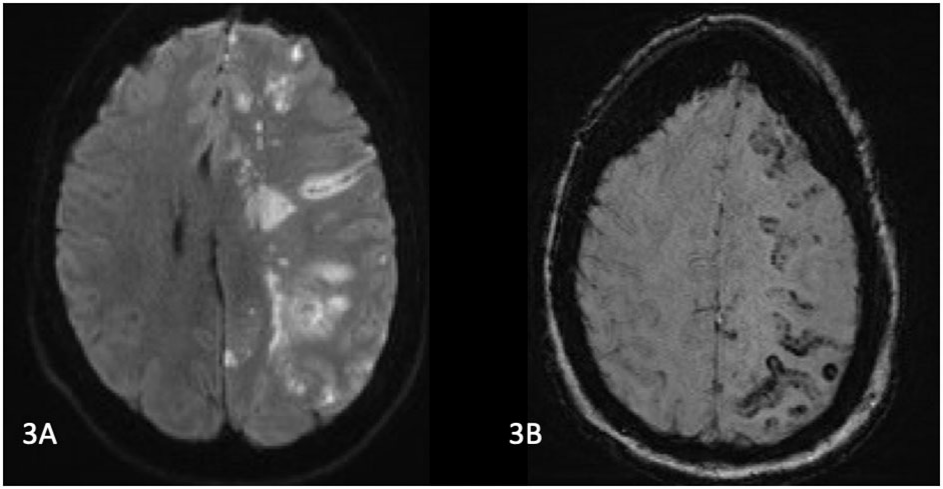
**(A)** MRI Brain on day 1 post operatively. Diffusion weighted images show left MCA territory infarction with a similar distribution of abnormalities on suspectibility weighted imaging) **(B)**. MCA, middle cerebral artery; DWI, diffusion weight imaging; ECR, endovascular clot retrieval.

Whilst in recovery, the patient was noted to have a GCS of 8 (E3V1M4). She had a severe right sided hemiparesis, left gaze deviation, with a NIHSS of 23. CT brain, angiogram of the brain/neck, and perfusion imaging revealed loss of grey-white differentiation in the left frontal and parietal lobes. There was no large vessel occlusion or significant perfusion lesion. Diffusion weighted MRI the next day revealed a left MCA territory infarction, with cortical, basal ganglia, and thalamic involvement, and associated susceptibility weighted imaging (SWI) changes (Figure 3). She was treated with a trial of hyperbaric oxygen (100% for 60 min) and dexamethasone. Transthoracic echocardiogram (TTE) revealed a PFO. The following day, the patient clinically deteriorated and was found to have significant cerebral oedema and midline shift, and an emergency decompressive hemicraniectomy was performed. After a period of monitoring without meaningful clinical improvement, she was palliatively managed and died in hospital.

## Discussion

Fat embolism syndrome (FES) is a clinical syndrome manifest by fat particles embolising into the systemic circulation and microcirculation. These emboli then cause damage within the microcapillaries, and may trigger a systemic immune response (1). Multiple different theories exist regarding the underlying pathophysiology of FES. The “mechanical theory” suggests that trauma causes the release of fat particles into the venous system, and then may enter the arterial system via cardiopulmonary shunts, or due to high pulmonary artery pressure (1, 2). The “biochemical theory” suggests that a stressed state within the body leads to systemic catecholamine release, and subsequently releases fat into the blood. Free fatty acids and activation of other inflammatory markers then lead to a systemic inflammatory response (1, 2). Overall, an inflammatory response causing an acute respiratory distress syndrome, diffuse petechial rash, and diffuse encephalopathy with associated MRI changes have been reported (3, 4). Patients typically develop symptoms within 24–72 h of the triggering event, presumed related to the inflammatory reaction (1). Neurological features often occur concurrently with respiratory distress (2).

Not all patients with fat embolism develop FES. FES is most commonly reported after orthopaedic trauma, due to the increased intramedullary pressure in the long bones in this setting (5). The incidence and prevalence of fat emboli in the context of orthopaedic procedures has been reported variably. One prospective review of patients undergoing long bone and pelvic fractures found that 4–10% of patients developed FES. An earlier review suggested a higher prevalence of 19% in patients presenting after orthopaedic trauma, and some reviews suggest a much lower incidence of <1% (4, 6). There are likely multiple reasons for this heterogeneity in identified incidence, including the possibility that FES may be under-recognised with concurrent significant traumatic injuries, and patients may variably fulfil diagnostic criteria. A review of 100 cases secondary to trauma, with or without orthopaedic injuries, described respiratory symptoms occurring in 75% of patients, and that approximately a third presented first with either neurological or dermatological or respiratory involvement. Any one of respiratory, neurological and dermatological involvement met their major criteria for the diagnosis of FES but they did not describe the incidence of all three triad components occurring (7).

Neuroimaging features in FES typically show diffuse and bilateral abnormalities. Diffuse cytotoxic oedema, often referred to as the “starfield pattern” on MRI, is the most commonly associated finding (8). A systematic review by Kuo et al. identified 5 primary patterns of MRI abnormalities, all of which correlate to FES being a primarily immunologic and microcirculation pathology (9). They found that patients most commonly have scattered embolic ischaemic features in the acute FES phase, which progresses to features of cytotoxic and vasogenic oedema in the subacute phases. Petechial haemorrhage may be seen in both the acute and chronic phases, and chronic sequelae, including cerebral atrophy, and features of demyelination, have been described. Radiodensity measurement of hypodensities within the brain parenchyma to correlate with the density of fat (variably reported between −30 and −100 HU), is not commonly reported, although the finding of a “hypodense artery,” has been more commonly reported in fat embolism associated LVO, and will be discussed further below (10–12).

In comparison to the well-established features of FES described above, all patients in our case series had features of LVO, warranting treatment with ECR and decompressive hemicraniectomy, which are not reported in FES patients. We performed a literature review to identify similar cases of fat embolism and LVO. Searches of Pubmed were independently performed by the first two authors. Medical search headings (MeSH) including “brain ischemia,” “stroke,” “ischemia,” “arterial occlusive diseases,” “ischemic stroke” were combined with “embolism, fat.” Six hundred ninety results were identified on Pubmed. On Embase, MeSH terms included “brain ischemia,” “cerebrovascular accident,” “ischemia,” combined with “fat embolism” identified 136 results. Where relevant publications were in other languages, attempts were made for English translations. Animal studies were excluded. All case reports and case series of patients who had identified large vessel occlusions on imaging, or who had a distribution of stroke in keeping with a large vessel occlusion were included where there was evidence of fat embolism. References of relevant articles were screened for additional cases.

Patients were excluded from our review where they underwent a recent procedure involving injection or removal of fat, including autologous fat filler procedures, and liposuction. The entity of filler induced cerebral embolism (FICE) has been well-reported and has been recently reviewed by Wang et al. FICE generally involves areas of extensive arterial supply and are known “danger zones” for intracranial emboli (13).

We identified 18 patients, in addition to the 3 reported in this series for the first time, and their characteristics are summarised in Supplementary Table 1. They ranged in ages from 25 to 91 years old. Nine patients were female, seven were male, and two did not have gender reported. Eight of the reported patients had undergone cardiac surgery, most commonly mitral valve replacement (6 patients) (11, 14–20). Six patients had orthopaedic procedures or preceding trauma, and three patients were deemed spontaneous (2, 10, 21–27). Of the spontaneous cases, one patient had a history of liposuction and gluteal augmentation 2 months prior, but this was presumed unrelated (2). One patient had LVO 9 days after Caesarean section, with multiple other embolic complications (28). Eleven of the 15 patients who did not have spontaneous LVO had onset of symptoms within 6 h of the presumed mechanism of injury (10, 11, 14, 15, 19–21, 23–25, 27). Four of the five patients who underwent PFO assessment were found to have a PFO (23–25, 27, 28). None of the patients had reported dermatologic manifestations, and only two patients had respiratory features (10, 28). With regards to treatment where it was available, one patient was treated with intravenous thrombolysis, eight patients underwent ECR or attempted ECR and three patients underwent decompressive hemicraniectomy. One patient also underwent (superficial temporal artery to middle cerebral artery) STA-MCA bypass in an attempt to bypass a right internal carotid artery occlusion (14). Of the patients with reported outcomes, 7 patients died within 3 months, 3 had mild persistent symptoms, and 3 had significant functional disability.

Our case series and literature review highlight key clinical, radiological and management considerations in LVO from fat embolism, in contrast to fat embolism syndrome (FES), which are summarised in Table 1. As highlighted above, the clinical presentation of the LVO cohort of patients was different, with most patients presenting within 6 h of the triggering event, in comparison to 24–72 h in FES patients. This might relate to some differences in the underlying pathophysiology. It is conceivable that in the context of PFO permitting intra-cardiac shunting, as was the case in patient 1 and 3 of our series, a sufficiently large fat vacuole could occlude a main branch in the Circle of Willis, with severe clinical symptoms occurring earlier due to mechanical occlusion. This is in contrast to FES, whereby the inflammatory cascade begins with free fatty acid release which causes a diverse range of systemic and central nervous system symptoms, with the pulmonary circulation possibly acting as a “buffer,” rather than the clinical expression of cerebral LVO (1).

**Table 1:** Difference in features between fat embolism syndrome and fat embolism related large vessel occlusion.

	**Fat embolism syndrome**	**Fat embolism related large vessel occlusion**
Precipitants	Most commonly orthopaedic trauma/procedures	Most commonly orthopaedic trauma/procedures, also seen post cardiac surgery (most commonly mitral valve replacement)
Symptom onset	Typically within 24–72 h	Most cases occurred within the first 6 h of the precipitant
Associated respiratory/dermatological manifestations	Typically present	Typically absent
Treatments	Conservative management	Thrombolysis, endovascular clot retrieval and decompressive hemicraniectomy may be considered depending on clinical and neuroimaging features
Prognosis	Typically good	High mortality and/or permanent disability

Other features of fat embolism related LVO also support the “mechanical theory” hypothesis. Changes to intramedullary pressure, tourniquet release and limb reperfusion are postulated to cause right atrial pressure to increase. This may in turn precipitate intracardiac shunting *via* a PFO of released fat emboli (29) in the context of orthopaedic instrumentation of the medullary canal and increase risk of stroke. Four of the five patients in our literature review and two patients in our series were noted to have PFO, compared to the population prevalence of PFO from autopsy studies of 27.4%. A retrospective cohort study showed that peri-operative ischaemic stroke in patients with PFO is 3.2% compared to 0.5% in patients without PFO, and whilst fat embolism is often mentioned as a cause of perioperative stroke, the risk is not quantifiable (30). The findings of fat embolism related LVO post cardiac surgery may be related to the sternal bone marrow being a source of lipid emboli, or the cardiopulmonary bypass apparatus, which may also reintroduce scavenged pericardial blood containing fat emboli (31).

Given the common mechanism of fat emboli underpinning both FES and LVO, it is likely that LVO strokes lie on the spectrum of clinical phenomena that can be seen with fat emboli regardless of source. Illustrative of this is patient 3 in our case series, whom had more typical findings of FES with intraoperative transient hypoxia and hypotension, but was found to have a single territory LVO infarction. Respiratory and dermatological features that are typically seen in FES are also not seen as frequently in fat embolism LVO patients. In our literature review and our cases, no patients had dermatological features, and only 2 had respiratory features. The presumed mechanism for respiratory and dermatological features relates to progressive venous congestion after the inflammatory response is triggered – we speculate that this phenomenon does not occur as frequently in LVO patients, and there may be less inflammatory response in these patients (32). This is also supported by their earlier presentations, and also by MRI findings. The finding of the hypodense artery sign has been consistently reported in the literature, with Hounsfield units (HU) of −30 to −100 reflecting intraarterial fat, and was used to assist in the diagnosis in our case series (11, 12). In FES, the end product of the inflammatory response is the typical diffuse “starfield pattern,” reflecting cytotoxic oedema (2). This was not seen in our case series, or commented on in the cases in the literature. The lack of respiratory or dermatological features is of relevance, as it may pose an additional challenge for the diagnosis of fat embolism associated LVO in the intraoperative or immediate post-operative state, where patients are also under the effects of anaesthesia and neurological assessment may be more challenging.

In FES, the overall mortality is 10%, and most patients return to baseline function (8, 33). In contrast, cases of LVO from fat emboli appear more neurologically severe compared to FES, with previous patient reports requiring decompressive hemicraniectomy for diffuse cerebral oedema (10, 15, 28, 34). Endovascular clot retrieval is also emerging as a treatment option for these cases (2, 14, 22–26, 28, 35). Successful endovascular clot retrieval has also been performed in large vessel occlusions due to fat embolism during liposuction (35). Techniques of endovascular clot retrieval vary in the devices used and include stent-retrievers, distal aspiration catheters, and proximal balloon occlusion catheters used alone or in combination. Selection of technique reflect site of occlusion, anatomical access, operator preference, and with clot composition one additional factor influencing first pass effect or final reperfusion success. Prospective assessment of clot composition can be based on density on NCCT, perviousness on post contrast studies, length, and MRI identification of red blood cell rich clot. Currently there is no consensus on procedural choice based on these findings, other than to suggest that thrombolysis may be less effective in the less common calcified, tumour or fat containing emboli, hence a lower threshold for proceeding with mechanical thrombectomy. Distal contact aspiration alone (ADAPT) OR used in combination with stent retrievers would be a favoured first line approach. Of the 7 reported patients who underwent reperfusion therapy (ECR or STA-MCA bypass, excluding the patient who had unsuccessful ECR), 5 survived with reasonable function (modified Rankin scale 3 or better from case descriptions), one patient died due lack of clinical improvement, and another died due to intracranial haemorrhage shortly after ECR (2, 14, 22–26). In comparison, of the patients who did not receive reperfusion therapy or received unsuccessful reperfusion therapy, five patients died, and two were functionally disabled (mRS 4) (10, 15, 17, 18, 21, 27, 28). The outcomes of 4 patients are unknown.

## Conclusion

To our knowledge, this is the largest case series of stroke from large vessel occlusion caused by fat embolism. We illustrate two cases of stroke from large vessel occlusions as a complication from orthopaedic surgery due to paradoxical cerebral fat embolism from PFO, and one case from cardiac sternotomy. In our first case, we were able to obtain histological confirmation by demonstrating the combination of adipocytes, bone marrow constituents and thrombus.

Fat emboli causing large vessel occlusion can be an important cause of acute neurological deterioration in the immediate post-operative period. In keeping with previous case reports, our case series highlights the importance of recognising large vessel stroke due to fat emboli post orthopaedic and cardiac surgery, and that patient outcomes appear poorer in comparison to fat embolism syndrome. It is likely that there is a clinical spectrum of FES which includes the patients presenting with LVO. Clinicians should be aware of this entity, and prompt neuroimaging should be performed when the diagnosis is suspected, as recanalisation treatments may improve clinical outcomes.

## Data Availability Statement

The original contributions presented in the study are included in the article/Supplementary Material, further inquiries can be directed to the corresponding author/s.

## Ethics Statement

Written informed consent was obtained from the individual(s) for the publication of any potentially identifiable images or data included in this article.

## Patient consent

Informed consent was obtained from all patients described in this case series. In circumstances where the patients could not express consent, informed consent was obtained from their legally appointed medical decision maker(s).

## Author Contributions

SO and SR contributed equally to writing the manuscript. PM contributed to writing the manuscript. BC, EH, GC, and PM curated the cases, imaging, and reviewed the manuscript. All authors contributed to the article and approved the submitted version.

## Conflict of Interest

The authors declare that the research was conducted in the absence of any commercial or financial relationships that could be construed as a potential conflict of interest.

## Publisher's Note

All claims expressed in this article are solely those of the authors and do not necessarily represent those of their affiliated organizations, or those of the publisher, the editors and the reviewers. Any product that may be evaluated in this article, or claim that may be made by its manufacturer, is not guaranteed or endorsed by the publisher.
