# Supplementary material for: Cerebral Large Vessel Occlusion Caused by Fat Embolism—A Case Series and Review of the Literature
Source: Front Neurol. 2021 Oct 13;12:746099. doi: 10.3389/fneur.2021.746099 (PMC8548632; doi:10.3389/fneur.2021.746099)
Supplement: Supplementary file 1 [file Table_1.docx]

| **Journal** | **Authors** | **Year of Publication** | **Age**  **Sex** | **Mechanism of injury** | **Time to Symptoms** | **Clinical features on assessment** | **Evidence of fat embolism** | **Other associated features (including cardiorespiratory or dermatological manifestations)** | **Imaging findings** | **Intervention** | **PFO** | **Outcome** |
| --- | --- | --- | --- | --- | --- | --- | --- | --- | --- | --- | --- | --- |
| Journal of Medical Imaging and Radiation Oncology (21) | Koh D, Nair G, et al | 2021 | 91F | Right hip hemiarthroplasty | Post-operative | Drowsiness limiting further assessment | Hypodense artery (-30 HU) | Not reported | Right MCA (M1 branch) occlusion | None | Not reported | Deceased |
| Surgical Neurology International (22) | Fowler J., Fiani B. et al | 2021 | 70M | Spontaneous | Unknown (Woke with symptoms) | Left side weakness and impaired coordination | Histopathology suggestive of fat embolism  Hyperdense artery (atypical finding) | Absent | Right ICA and distal MCA occlusion | ECR | Not reported | Mild left hemiparesis, discharged home, mRS 1 |
| BMJ Neurology Open(14) | Van Der Veken J., Presti A et al. | 2020 | 69 | Mitral valve replacement | Post procedure | Left hemiparesis | Hypodense artery | Absent | Right ICA occlusion | ECR, STA-MCA bypass | Not reported | Able to walk independently, dense left upper limb weakness |
| Intensive Care Medicine(23) | Maier B, Badat N et al. | 2019 | 56M | Right knee replacement (spinal anaesthesia) | Immediately post procedure | Right hemiplegia / aphasia | Hypodense artery (-33 HU) | Not reported | Left MCA occlusion | ECR | Yes | Recovered ‘well’ |
| Neurology(24) | Gopal M, Thakur G et al. | 2019 | 70F | Right total hip arthroplasty | Post procedure | Unresponsive, hypotensive, hypoxic | Hypodense artery | Not reported | Thrombus at tip of basilar artery | ECR | Yes | Deceased |
| Medicine(2)* | Lee H, Park J., et al | 2019 | 80M | Spontaneous (liposuction and gluteal augmentation 2 months prior presumed unrelated) | NA | Dizziness, diplopia, leg weakness, mild dysarthria | Histological evidence | Absent | Distal basilar artery occlusion | ECR | Unknown | Gait disturbance at 1 year follow-up |
| Emergency Medicine and Critical Care(28) | Ion I, Parvu T, et al | 2018 | 25F | Caesarean section delivery | 9 days | Aphasia, right hemiplegia | Hypodense artery | Acute respiratory distress syndrome 2 days later  Bilateral pulmonary infiltrates on imaging  Multiple splenic, hepatic, and omental infarctions, ischaemic leg | Left M1 MCA occlusion | Failed ECR  Decompressive hemicraniectomy | No | mRS 4 at 3 month review |
| Practical Neurology(15) | Avila JD | 2017 | Mid 70s F | Aortic valve replacement and single coronary artery bypass grafting | Immediately post procedure | Left hemiplegia, right gaze deviation | Hypodense artery (-80 HU)  Autopsy confirmed – mature adipose tissue | Not reported | Distal right MCA M1 occlusion | Decompressive hemicraniectomy | Unknown | Significant disability, deceased after 1 month |
| Applied Radiology(16) | Sinha N, Fertakos R, et al | 2014 | Not reported | Mitral valve replacement | Within 24 hours | Left hemiplegia | Hypodense artery | Not reported | Right MCA occlusion | None | Unknown | Unknown |
| Case Reports in Orthopaedics(25) | Piuzzi NS, Zanotti G., et al | 2014 | 80 M | Revision hip surgery | Post procedure | Left facial weakness, left hemiplegia, sensory impairment | Hypodense artery | Absent | Right MCA occlusion | ECR, successful recanalization, complicated by intracranial haemorrhage | Yes | Died at 3 months |
| Journal of Neurosurgery(10) | Kellogg R, Fontes R et al | 2013 | 58F | Intertrochanteric fracture R femur | 6 hours after admission | Generalised tonic-clonic seizure, left hemiplegia | Multiple hypodensities (-90 HU), primarily in the right cerebral hemisphere* | Respiratory symptoms requiring intubation | Likely right MCA | Decompressive hemicraniectomy | Unknown | mRS 4 at 6 month review |
| Journal of the Belgian Society of Radiology (17) | Bogdan J, Rommel D | 2013 | 77M | Mitral valve replacement and coronary artery bypass grafting | Day 2 | Left hemiplegia, facial droop | Hypodense artery | Not reported | Right MCA occlusion | None | Unknown | Deceased |
| The Neurohospitalist (11) | Malik | 2012 | 88F | Mitral valve replacement | Postoperative | Right hemiplegia | Hypodense artery (-50 HU) | Not reported | Left MCA occlusion | Unknown | Unknown | Unknown |
| Neurology(18) | Wang Y, Fu J, Lai P | 2009 | 52F | Mitral valve replacement | Day 2 | Left hemiplegia | Hypodense artery (-83 HU) | Not reported | Distal right ICA occlusion | None | Unknown | Deceased |
| Archives of Neurology(26) | Grunwald IQ, Bose A, et al | 2009 | 41M | Spontaneous | Presented 45 minutes post onset | Right hemiplegia, aphasia | Histological evidence | Absent | Left MCA occlusion | Intravenous thrombolysis, followed by ECR | Unknown | Persisting mild dysarthria and dysphasia |
| Neurocritical Care(19) | Abend N, Levine J | 2007 | 44M | Aortic valve repair | Day 0 post procedure | Unknown | Hypodense artery | Not reported | Proximal right MCA occlusion | Unknown | Unknown | Unknown |
| American Journal of Neuroradiology(20) ** | Lee T, Barlett E et al, | 2005 | 78F | Mitral valve replacement | Immediately post procedure | Right hemiparesis, aphasia, decreased GCS | Hypodense artery (-25 HU) | Not reported | Left MCA M1 occlusion | None | Unknown | Unknown |
| Journal of Cerebrovascular Diseases(27) | Van Oostenbrugge RJ, Freling G. et al | 1996 | 40F | Bilateral intramedullary fixation to prevent pathological fractures | Post procedure | Reduced GCS, right hemiplegia | Histological evidence | Transient intraoperative hypotension and ECG changes | Left MCA occlusion | None | Yes | Died 40 hours later |

*This patient did not have definite evidence of large vessel occlusion, however, the presence of hypodensities primarily within the right cerebral hemisphere, of a density consistent with fat embolus (-90 HU) and the subsequent development of a malignant MCA like syndrome requiring decompressive hemicraniectomy makes it likely there was a large vessel occlusion.

**Lee et al presented 2 cases, one of which was a filler induced cerebral embolism and not described here

Table 1: Characteristics of patients with large vessel occlusions causing fat embolism.

*Abbreviations: ECR – endovascular clot retrieval, GCS – Glasgow Coma Scale, MCA – middle cerebral artery, ICA – internal carotid artery, HU – Hounsfield units, mRS – modified Rankin scale, STA-MCA – superficial temporal artery to middle cerebral artery, M – male, F – female*
